# Effects of Platelet-Rich Plasma (PRP) on a Model of Renal Ischemia-Reperfusion in Rats

**DOI:** 10.1371/journal.pone.0160703

**Published:** 2016-08-23

**Authors:** Oriol Martín-Solé, Joan Rodó, Lluís García-Aparicio, Josep Blanch, Victoria Cusí, Asteria Albert

**Affiliations:** 1 Unit of Pediatric Urology, Department of Pediatric Surgery, Hospital Sant Joan de Déu, Universitat de Barcelona, Barcelona, Spain; 2 Department of Pediatric Radiology, Hospital Sant Joan de Déu, Universitat de Barcelona, Barcelona, Spain; 3 Department of Pathology, Hospital Sant Joan de Déu, Universitat de Barcelona, Barcelona, Spain; 4 Department of Pediatric Surgery, Hospital Sant Joan de Déu, Universitat de Barcelona, Barcelona, Spain; Universidade Federal de Sao Paulo, BRAZIL

## Abstract

Renal ischemia-reperfusion injury is a major cause of acute renal failure, causing renal cell death, a permanent decrease of renal blood flow, organ dysfunction and chronic kidney disease. Platelet-rich plasma (PRP) is an autologous product rich in growth factors, and therefore able to promote tissue regeneration and angiogenesis. This product has proven its efficacy in multiple studies, but has not yet been tested on kidney tissue. The aim of this work is to evaluate whether the application of PRP to rat kidneys undergoing ischemia-reperfusion reduces mid-term kidney damage. A total of 30 monorrenal Sprague-Dawley male rats underwent renal ischemia-reperfusion for 45 minutes. During ischemia, PRP (PRP Group, *n* = 15) or saline solution (SALINE Group, *n* = 15) was administered by subcapsular renal injection. Control kidneys were the contralateral organs removed immediately before the start of ischemia in the remaining kidneys. Survival, body weight, renal blood flow on Doppler ultrasound, kidney weight, kidney volume, blood biochemistry and histopathology were determined for all subjects and kidneys, as applicable. Correlations between these variables were searched for. The PRP Group showed significantly worse kidney blood flow (p = 0.045) and more histopathological damage (p<0.0001). Correlations were found between body weight, kidney volume, kidney weight, renal blood flow, histology, and serum levels of creatinine and urea. Our study provides the first evidence that treatment with PRP results in the deterioration of the kidney’s response to ischemia-reperfusion injury.

## Introduction

Renal ischemia with reperfusion injury is a major cause of acute renal failure [1], and is often seen in situations such as partial nephrectomy, renal transplantation, renal trauma, hypovolemia, hypotension, sepsis, dehydration, acute tubular necrosis, shock with multiorgan failure and surgical reconstruction procedures that require renal artery occlusion for an extended period [2]. This condition causes the death of renal epithelial cells and contributes to the delayed recovery of kidney function after transplantation and the subsequent chronic renal hypoxia is an important mechanism in the development of tubulointerstitial fibrosis and the progression of chronic renal disease [3]. Renal ischemia-reperfusion has been studied in small animal models [4–6], focusing primarily on renal transplantation, with the aim of minimizing damage and improving graft survival. Many of the drugs and growth factors used in animal models of renal ischemia-reperfusion have demonstrated their effectiveness [7, 8], but their use in clinical practice has been limited by their high cost.

Platelet-rich plasma (PRP), first described by Marx et al. [9] in 1998, is an autologous product rich in growth factors obtained from a blood sample, which is centrifuged to isolate the platelet-rich supernatant. PRP needs to be activated by products such as calcium chloride or fibrinogen [10].

Many growth factors are released locally by PRP up to three weeks after application: platelet-derived growth factor (PDGF) which stimulates the formation of Type I collagen and promotes angiogenesis; transforming growth factor beta 1 (TGF-β1) which stimulates the proliferation and differentiation of mesenchymal stem cells and the synthesis of Type I collagen, also promoting angiogenesis; epidermal growth factor (EGF) which stimulates granulation tissue; vascular endothelial growth factor (VEGF) which induces chemotaxis and the proliferation of endothelial cells, promoting angiogenesis, vascular hyperpermeability and renal stem cell differentiation; basic fibroblast growth factor (b-FGF); insulin-like growth factor (IGF); platelet factor 4 (PF4); adenosine triphosphate (ATP); adenosine diphosphate (ADP); Angioprotein-2; Fibronectin; Osteocalcin; Serotonin; and Thrombospondin-1 (TSP-1), among others [11, 12].

Several of these growth factors, mainly EGF, IGF, TGF- β1 and VEGF, are released during renal ischemia-reperfusion. EGF is a potent promoter of growth in the renal tubular cells that attenuates tubular necrosis [13]. IGF is a hormone that ameliorates acute tubular necrosis [14]. TGF- β1 increases antiapoptoic Bcl-2 expression, maintains epithelial homeostasis and protects renal cells from apoptosis [15, 16]. VEGF protects peritubular endothelium, induces the proliferation of tubular epithelial cells, promotes angiogenesis and accelerates renal recovery after ischemia [17–19]. All of these growth factors are known to be released by PRP, so it could be expected that the application of PRP to an ischemic kidney would improve its recovery.

Platelet-rich plasma has demonstrated its efficacy in numerous studies in the field of plastic and maxillofacial surgery, dentistry and orthopedics, as a regenerative and angiogenic product acting locally to enhance bone, muscle, tendon, cartilage and skin growth, among other effects [20–23]. In fact, it is routinely used in some centers to treat bone fractures, as an aid in dental implants and prosthesis, and to treat diabetic ulcers and dry eye in Sjögren’s syndrome, among other applications. Furthermore, PRP has also proven useful in end-to-end intestinal and tracheal anastomosis, improving blood flow and scar strength [24, 25].

The major advantage of PRP over other ways of administering growth factors is that it is an inexpensive product, easy to obtain, and when it is autologous there is no risk of rejection or immune reaction. Furthermore, an antimicrobial action of PRP containing leukocytes has been described, suggesting a low risk of infection [26, 27].

Much research has been done on the effects of drugs and growth factors on renal ischemia-reperfusion [7, 8]. However, there are no publications on the effects of PRP on the kidney.

If the results proved positive, the application of PRP before a surgical procedure requiring renal ischemia, such as renal transplantation or partial nephrectomy, would improve renal prognosis. Although PRP has proven useful as a regenerative product and it releases growth factors known to improve the kidney after ischemia, there are no publications studying the effects of PRP on the kidney. Therefore, the aim of this study is to investigate whether PRP protects the kidney from ischemia-reperfusion damage.

## Materials and Methods

### Animals

A total of 45 Sprague-Dawley male rats weighing 200 to 225g were acquired from Charles River Laboratories, France, and acclimatized at our animal care facility for two weeks. All the animals were kept in separate cages under controlled conditions of temperature and humidity, with a light/dark cycle of 12/12 hours, and were allowed food and water *ad libitum*. Animals were randomized into 3 groups of 15 rats: Group 0 (blood donors sacrificed to prepare PRP), the PRP Group (renal ischemia treated with PRP), and the SALINE Group (renal ischemia treated with vehicle only–saline-).

### Ethics Statement

All animal studies were carried out in accordance with and after receiving the approval of the Universitat de Barcelona Ethical Committee for Animal Experimentation, and in accordance with the European Parliament and Council normative 2010/63/UE (22nd September 2010) on the protection of animals used for scientific purposes. Humane endpoints used during the animal survival study were: rapid weight loss of >20% of body weight, surgical complications unresponsive to medical intervention, poor physical appearance with abnormal behavior (reduced mobility, unconsciousness or self-mutilation), severe respiratory distress, neurological signs, frank bleeding from any orifice and impaired ambulation which prevented animals from reaching food or water. Animals that reached humane endpoints were euthanized through complete exsanguination via cardiac puncture under general anesthesia with inhaled 2% isoflurane.

### PRP preparation and activation

Rats in Group 0 were anesthetized by inhalation of 2% isoflurane, and 5-10mL of blood was drawn via cardiac puncture. Each blood sample was introduced into vacuum tubes containing EDTA (K2). One milliliter of each sample was used for the blood count and the remainder was used to prepare PRP. Blood cell elements were separated using a laboratory centrifuge at 200g for 15 minutes at 20°C, resulting in two basic components: red blood cells at the bottom (4-8mL) and PRP on top, yielding 1–2.5mL. The red blood cells were decanted from the remaining sample. One milliliter of PRP was separated for blood count when PRP volume was more than 1.8mL. PRP was classified according to the PAW Classification System [28]. PRP was activated immediately before its application, with CaCl_2_ 10% (0.8mL of PRP + 0.2mL of CaCl_2_ 10%).

### Renal ischemia-reperfusion injury model

Rats in the PRP and SALINE Groups were anesthetized by inhalation of 2% isoflurane. Using a right abdominal incision, the right renal pedicle was ligated and divided, and a right nephrectomy was performed. The right kidney was cut longitudinally and fixed in formalin. Using a left abdominal incision, the left renal pedicle was clamped for 45 minutes with a microaneurysm clamp. The renal warm ischemia time (WIT) used in previously published studies of renal ischemia-reperfusion in rats varied between 30 and 120 minutes, with a survival rate of 90% and 0% at 30 days after reperfusion respectively. According to the literature, a renal WIT duration of 45 to 75 minutes is most commonly chosen because that WIT range allows for intermediate survival. We chose 45 minutes of renal ischemic time, with a known survival rate of 100% at 7 days and 85% at 30 days [4]. After removing the clamp, the left kidney was inspected for restoration of blood flow. The abdomen was closed in 2 layers on each side, and 1mL of 0.2% mepivacaine was injected into each wound. Subcutaneous injections of dexketoprofen trometamol were administered as analgesia at a dose of 10mg/kg before the start of the first surgical intervention and then every 12 hours during the first 4 days, after which no additional analgesic treatment was required. Animal wellbeing was monitored every eight hours. Humane endpoints were applied during survival analysis as set out in the Ethics Statement section. Rats were euthanized seven days after the first procedure through complete exsanguination via cardiac puncture under inhalatory anesthesia with 2% isoflurane; the blood was used for biochemical tests. Left kidneys were harvested, cut longitudinally and fixed in formalin. Total reperfusion time was the seven days between renal ischemia and euthanasia. Since it is well known than PRP releases growth factors mainly during the first week after its application [11], and we wanted to study its medium-term effects on the kidney, we decided to set seven days as the end point of our study.

### Application of platelet-rich plasma or saline in kidneys during ischemia

**Fig 1:**
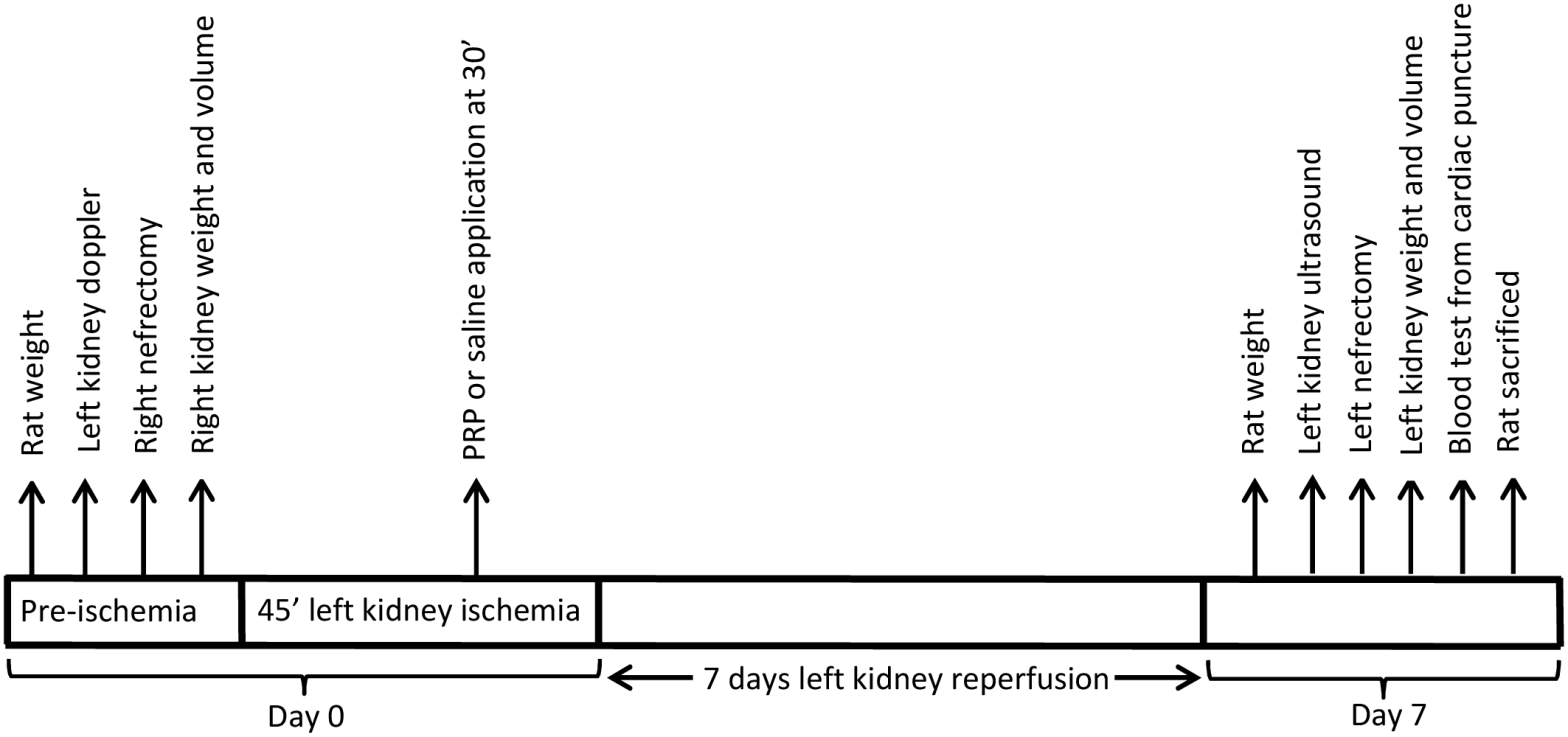
Experimental protocol.

Activated PRP was injected into the kidneys of the PRP Group rats, exactly 30 minutes into ischemia. Five subcapsular punctures were performed (anterior, posterior, lateral, upper pole and lower pole), distributing the whole amount of activated PRP (1mL) over the renal surface. The SALINE Group rats were injected with 1mL of saline following the same protocol. The dose of PRP used in other experimental studies varies greatly, ranging from 0.05mL to 5mL [21, 29–34]. Since there is no guidance in the literature on PRP dosing in rat kidneys, a pilot study was conducted on four rats before starting the present study. A volume of 1mL proved to be the required amount to cover the whole subcapsular surface of the kidney. The experimental protocol is shown in Fig 1.

### Measurement of rat weight, kidney weight and kidney volume

All rats were weighed before the procedure (day 0) and before harvesting (day 7). The weight and volume of right and left kidneys were measured after nephrectomies and before formalin fixation. Renal density was calculated.

### Ultrasound study

Left kidney ultrasound was performed before ischemia (day 0) and on the seventh day after ischemia. The left renal artery was identified on Doppler ultrasound. The following parameters were calculated: pulsatility index (PI), resistive index (RI), maximum velocity (Vmax), end diastolic velocity (EDV), systolic/diastolic ratio (S/D), and time to reach maximum flow peak (TPMx).

### Histological study

Right and left kidneys were cut longitudinally and fixed in formalin for 12 to 24 hours after nephrectomies. Specimens were embedded in paraffin, and semi-thin sections (1μm) were prepared and stained with hematoxylin and eosin (H&E). A set of parallel sections were processed with PAS staining, and served to document interstitial edema and the possible ablation of epithelial cells from the basement membranes. All the samples were labeled with random numbers for blind analysis. All analyses were performed blind by the same researcher (OMS), with the assistance of VC. Ten high power fields (magnification 600X) per sample were examined. A semi-quantitative study was performed with the method used by Martin Alexander [35] where a score of 0 was given when no deviations from the normal pattern were found, 1 for mild deviations, 2 for medium and 3 for severe damage. Histopathological changes were assessed using the following parameters: interstitial edema, dilation of the peritubular capillaries, vacuolization, ablation of tubular epithelium from the basement membrane, ablation of the brush border from the epithelium of the proximal tubuli, and cell death (cell swelling and defragmentation of cell nuclei). The addition of these values results in a score between 0 and 18. Additionally, 25 randomly chosen glomeruli from the tissue sections of each group were studied, and distal and proximal tubular cells around the glomeruli were analyzed for vesicles under high power fields (600X magnification) and a score of 0 to 4 was given for each glomerulus: 0 = none; 1 = <25%; 2 = 25–50%; 3 = 50–75%; 4 = >75% of the epithelial cells displaying vesicles. The result is reached by the addition of the scores for each glomerulus, with the maximum possible score being 100.

### Biochemical study

Serum levels of creatinine and urea were determined in blood from cardiac puncture on the seventh day in both groups. After centrifuging the samples, serum was separated and kept at −80°C until analyzed with an automated system (ARCHITECT ci8200, Abbott Diagnostics) after centrifuging at 20°C.

### Survival

The time and cause of each death occurring before the end of the study was recorded and analyzed.

### Statistical analysis

IBM SPSS Statistics 22.0 software was used to analyze the variables. For quantitative variables unpaired and paired Student’s *t* tests were used. For categorical variables, Fisher’s exact test was used. For correlations, Pearson *r* coefficient was used. For survival analysis, Kaplan-Meier test was used. All tests were two-sided tails. Results are shown as mean ± standard deviation, or median and interquartile range (IQR). Statistical significance was assumed at p<0.05.

## Results

### PRP analysis and classification

Blood from donor rats (Group O) was analyzed before and after obtaining PRP. Platelet rich plasma contained a mean of 1282000 ± 363000 platelets/μL, 1.7 times the mean blood platelet level (747000 ± 236000 platelets/μL), p<0.001. White blood cell count was 8150 ± 2500/μL in plasma and 5300 ± 3600/μL in PRP (p = 0.002); there were 390 ± 220 neutrophils/μL in plasma, and 328 ± 212 neutrophils/μL in PRP, p = 0.364. According to the PAW classification system proposed by DeLong et al. [28], this PRP is classified as P4-x-Bβ (*P4* meaning that there are >1250000 platelets/μL; *x* meaning that an exogenous method of activation is used, such as CaCl_2_; *B* meaning that the total white blood cell count is equal to or below baseline; and β meaning that the neutrophil count is equal to or below baseline).

### Weight loss in the week after renal ischemia

There were no differences in body weight before ischemia between the groups (mean in the PRP Group 344 ± 28g vs. 347 ± 38g in the SALINE Group, p = 0.816). However, there was a statistically significant weight loss in both groups one week after ischemia (348 ± 33g pre-ischemia vs. 320 ± 41g seven days post-ischemia, p<0.0001), though the difference between groups was not significant: mean weight loss in the PRP Group was 30 ± 23g, while in the SALINE Group it was 26 ± 29g (p = 0.728).

### Increase in the kidney weight and density in the week after renal ischemia

The weight and density of the right, non-ischemic, kidneys was similar in both groups: mean weight in the PRP Group = 1.35 ± 0.11g vs. 1.33 ± 0.07g in the SALINE Group, p = 0.654; mean density in the PRP Group was 0.90 ± 0.07 g/mL vs. 0.90 ± 0.06 g/mL in the SALINE Group, p = 0.984. Kidney weight and density were higher seven days after ischemia: 1.34 ± 0.09g vs. 2.46 ± 0.58g, p<0.0001; 0.90 ± 0.06g/mL vs. 0.97 ± 0.06 g/mL, p = 0.001. However, there were no differences between groups seven days after ischemia, with a mean kidney weight of 2.54 ± 0.69g in the PRP Group vs. 2.38 ± 0.47g in the SALINE Group (p = 0.455), and a mean density of 0.98 ± 0.05g/mL in the PRP Group vs. 0.95 ± 0.06 g/mL in the SALINE Group (p = 0.229).

### PRP treatment reduces renal artery Vmax one week after renal ischemia

We observed an increase in the pulsatility index (PI), resistive index (RI), and systolic/diastolic ratio (S/D), and a decrease in the end diastolic velocity (EDV) decreased one week after ischemia in both groups (Table 1). There were no differences in maximum velocity (Vmax) before and after ischemia. However, when comparing both groups after ischemia, differences were found in Vmax (73.08 ± 20.31cm/s in the PRP Group vs. 87.1 ± 14,15cm/s in the SALINE Group, p = 0.045), but not in other Doppler parameters (Table 2).

**Table 1:** Renal blood flow before and one week after ischemia-reperfusion.

	Pre-ischemia	Seven days post-ischemia	Mean differences	p-value^a^
PI	1.12 (±0.27)	1.39 (±0.48)	0.27	0.01*
RI	0.66 (±0.08)	0.71 (±0.11)	0.05	0.04*
Vmax (cm/s)	82.41 (±11.22)	80.87 (±18.24)	-1.53	0.71
EDV (cm/s)	28.80 (±8.81)	22.97 (±9.73)	-5.83	0.03*
S/D	3.07 (±0.86)	4.18 (±2.23)	1.12	0.02*
TP Max (ms)	49.93 (±10.40)	43.36 (±11.40)	-6.57	0.04*

PI, pulsatility index; RI, resistance index; Vmax, maximum velocity; EDV, end diastolic velocity; S/D, systolic/diastolic ratio; TP Max, time to reach maximum flow peak.

^a^ Paired Student’s *t* test. Values expressed as mean (±SD).

* p<0.05.

**Table 2:** Renal blood flow one week after ischemia-reperfusion.

	PRP Group	SALINE Group	Mean differences	p-value^a^
PI	1.43 (±0.55)	1.37 (±0.44)	-0.05	0.778
RI	0.72 (±0.13)	0.71 (±0.10)	-0.01	0.815
Vmax (cm/s)	73.08 (±20.31)	87.10 (±14.15)	14.02	0.045*
EDV (cm/s)	20.41 (±11.24)	25.02 (±8.16)	4.61	0.228
S/D	4.66 (±3.02)	3.80 (±1.32)	-0.86	0.33
TP Max (ms)	39.10 (±12.50)	46.76 (±9.52)	-7.66	0.082

PI, pulsatility index; RI, resistance index; Vmax, maximum velocity; EDV, end diastolic velocity; S/D, systolic/diastolic ratio; TP Max, time to reach maximum flow peak.

^a^ Unpaired Student’s *t* test. Values expressed as mean (±SD).

* p<0.05.

### More histological damage is observed in PRP Group than in SALINE Group after ischemia-reperfusion injury

**Fig 2:**
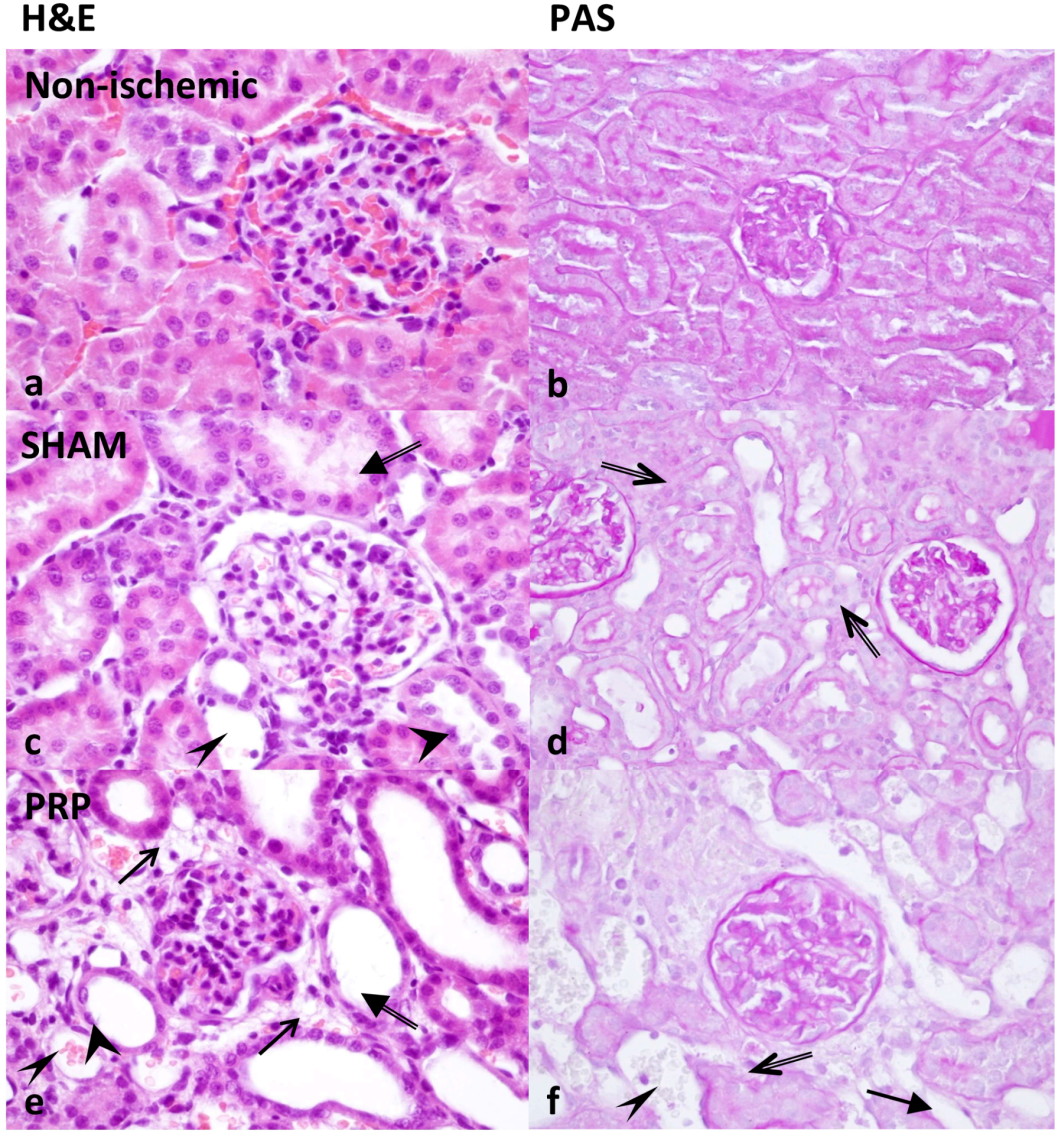
Representative images of the renal cortex after H&E staining (a, c, e) and after PAS (b, d, f). *Arrows* in **e** show vesicles in epithelial cells of the tubuli. *Thin arrowheads* in **c, e, f** point at peritubular capillary dilatation. *Arrow with broad head* in **f** shows interstitial edema. *Double-line arrows* in **d and f** show ablation of the tubular epithelium from the basement membrane. *Double-line arrows with broad heads* in **c, e** show loss of the brush border, and *broad arrowheads* show tubular epithelial cells with defragmented nuclei.

Ischemia-reperfusion injury produced histological damage to all kidneys when compared with non-ischemic kidneys (Table 3), but histological damage was significantly increased in the PRP Group compared to the SALINE Group (Table 4). Representative images of the renal cortex after H&E and PAS staining are shown in Fig 2.

**Table 3:** Histological damage. Non-ischemic vs. ischemic kidneys.

	Non-ischemic kidneys	Ischemic kidneys	p-value^a^
Interstitial edema (0–3)	0 (0–0)	2 (1–2)	<0.001*
Dilation of the peritubular capillaries (0–3)	0 (0–0)	2 (1–3)	<0.001*
Vacuolization (0–3)	0 (0–1)	2 (2–3)	<0.001*
Ablation of tubular epithelium from the basement membrane (0–3)	0 (0–0)	2 (1–3)	<0.001*
Ablation of the brush border of the proximal tubuli (0–3)	0 (0–0)	2 (1–3)	<0.001*
Cell death (0–3)	0 (0–0)	2 (1–2)	<0.001*
Total (0–18)	0 (0–1)	11.5 (7–14.75)	<0.001*
% Vesicles (0–100)	2 (0–5)	39 (25–68.25)	<0.001*

Values expressed as median (IQR).

^a^ Paired Student’s *t* test.

* p<0.05.

**Table 4:** Histological damage one week after ischemia-reperfusion.

	PRP Group	SALINE Group	p-value^a^
Interstitial edema (0–3)	2 (2–3)	1 (1–1)	<0.001*
Dilation of the peritubular capillaries (0–3)	3 (2–3)	1 (1–2)	<0.001*
Vacuolization (0–3)	2 (2–3)	2 (2–2)	0.045*
Ablation of tubular epithelium from the basement membrane (0–3)	2 (2–3)	1 (1–1)	<0.001*
Ablation of the brush border of the proximal tubuli (0–3)	2 (2–3)	1 (1–2)	<0.001*
Cell death (0–3)	2 (2–3)	1 (1–2)	<0.001*
Total (0–18)	13 (12–17)	7 (7–10)	<0.001*
% Vesicles (0–100)	54 (43–90)	26 (23–35)	0.001*

Values expressed as median (IQR).

^a^ Unpaired Student’s *t* test.

* p<0.05.

### Evaluation of kidney function after ischemia-reperfusion injury

The mean serum level of creatinine seven days after ischemia was 1.00 ± 0.87mg/dL in the PRP Group and 0.76 ± 0.78mg/dL in the SALINE Group, with no statistical difference (p = 0.452). Serum level of urea at seven days after ischemia was 94.8 ± 81.55mg/dL in the PRP Group and 83.3 ± 85.6mg/dL in the SALINE Group, but there was no statistical difference (p = 0.727). The percentage of rats that reached a level of serum creatinine over 1.00mg/dL on day seven was 33% in the PRP PRP vs. 13% in the SALINE Group, although this difference was not statistically significant (p = 0.388).

### Survival analysis

**Fig 3:**
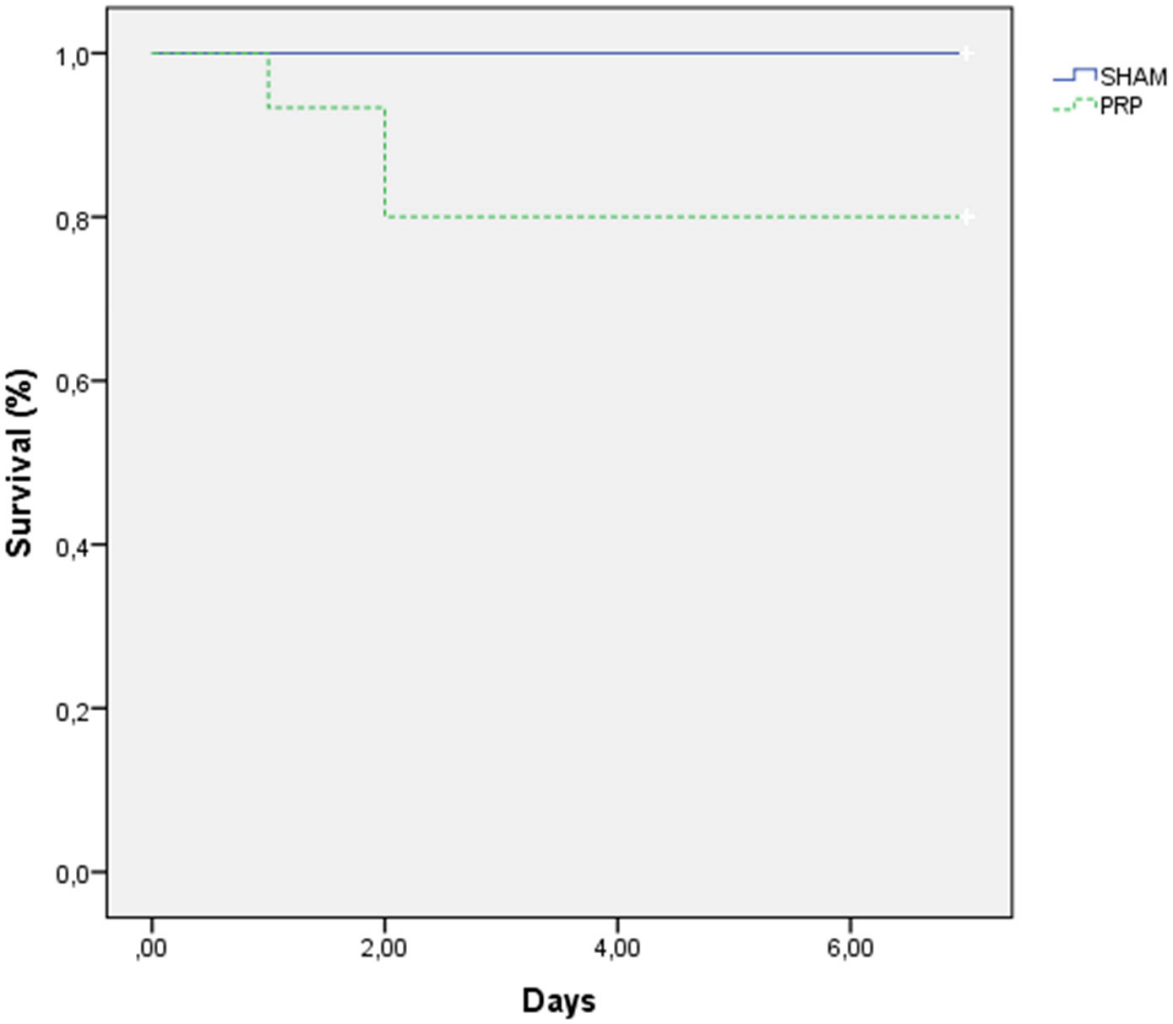
Survival function: Kaplan-Meier graph. p = 0.073.

Three rats died before the end of the study in the PRP Group (one on day two, two on day three); no rats died in the SALINE Group. The rat that died on day two showed signs of severe respiratory distress and reduced mobility, and was euthanized. The two rats that died on day three died unexpectedly, probably due to renal failure secondary to either the ischemia-reperfusion protocol or to renal postoperative complications of the remaining kidney. The results are shown in Fig 3. Although there was no statistical difference between groups according to the Kaplan-Meier analysis (p = 0.073), the difference was approaching significance.

### Correlation between histological damage scores, biochemistry, renal blood flow and kidney weight and volume after ischemia-reperfusion injury

Some interesting correlations were found between kidney weight, kidney volume, renal blood flow, histology and kidney function. There was a strong correlation between histological damage scores and biochemistry (serum levels of creatinine and urea), between renal blood flow and histological damage scores, between renal blood flow and biochemistry (serum levels of creatinine and urea), and between kidney weight, kidney volume and both biochemistry (serum levels of creatinine and urea) and histological damage. The results are shown in Table 5.

**Table 5:** Correlations between variables.

Variables correlated		Pearson r coefficient	p-value
Histology score	Creatinine	0.743	<0.001*
Histology score	Urea	0.736	<0.001*
% Vesicles (histology)	Creatinine	0.866	<0.001*
% Vesicles (histology)	Urea	0.820	<0.001*
Histology score	TP Max	-0.476	0.012*
Histology score	Vmax	-0.587	0.001*
% Vesicles (histology)	TP Max	-0.490	0.009*
% Vesicles (histology)	Vmax	-0.533	0.004*
% Vesicles (histology)	EDV	-0.414	0.032*
Creatinine	TP Max	-0.481	0.011*
Creatinine	V Max	-0.586	0.001*
Urea	TP Max	-0.505	0.007*
Urea	Vmax	-0.528	0.001*
Body weight 7 days after ischemia	Creatinine	-0.489	0.01*
Body weight 7 days after ischemia	Urea	-0.496	0.009*
Body weight 7 days after ischemia	% Vesicles (histology)	-0.392	0.043*
Kidney weight 7 days after ischemia	Creatinine	0.720	<0.001*
Kidney weight 7 days after ischemia	Urea	0.705	<0.001*
Kidney weight 7 days after ischemia	Histology score	0.406	0.029*
Kidney weight 7 days after ischemia	%Vesicles (histology)	0.497	0.006*
Kidney volume 7 days after ischemia	Creatinine	0.697	<0.001*
Kidney volume 7 days after ischemia	Urea	0.683	<0.001*
Kidney volume 7 days after ischemia	% Vesicles (histology)	0.442	0.016*

Vmax, maximum velocity; TP Max, time to reach the maximum flow peak.

* p<0.05.

## Discussion

In complete contrast to our initial expectations, our results show a deterioration in the quality of the kidneys studied instead of tissue regeneration. This was observed directly in the histological study and indirectly by ultrasound renal flow parameters, serum levels of creatinine and urea, kidney weight, kidney volume and survival. Although not all the results are statistically significant, there is a trend in all parameters towards a worsening of the organs. The fact that so many variables correlate suggests that a larger sample size would increase the number of variables reaching statistical significance.

Although most studies point to PRP as promoting tissue regeneration, some authors are less optimistic about its effects [36–38] and call for caution. In fact, despite the increasing number of articles published about PRP, most are actually basic science studies, animal studies, small case reports, reviews or opinion articles. PRP application has demonstrated major benefits in basic science, animal models and some low level evidence studies, but few clinical studies with a high level of evidence have reported similar results. In fact, there is a lack of randomized controlled trials studying the clinical effect of PRP and providing a high level of medical evidence when considering its potential benefits. The number of participants in studies tends to be small and the majority of studies are underpowered. Furthermore, studies examining the effects of PRP rarely have used standardized techniques. PRP is an acronym encompassing products with different platelet counts, obtained by different procedures. Each product differs in aspects that could markedly influence its clinical application, such as the blood volume harvested, the use of anticoagulant, the number and speed of centrifugation, the volume of PRP obtained and administered, activation method, the integrity of platelets, cryopreservation, and the presence of other cells, among other considerations. [39, 40] This fact makes it very difficult to conduct a systematic review or meta-analysis [22].

There are numerous studies in the literature illustrating the effects of PRP on skin, muscle, ligaments, bone, bowel, peripheral nerves, trachea, ovary, heart, liver, brain and cornea [20–25, 29, 41–44]. However, to the best of our knowledge, this is the first study to evaluate the effects of PRP on the kidney. Finding the best way to apply PRP to a solid organ wrapped in a capsule proved to be challenging. It is well known that the application of PRP should be local and not systemic. Until now it has been administered as an injected fluid, as a clot or as part of a scaffold [45]. As we were testing its effects on a solid organ, subcapsular injection seemed to be the most effective way to deliver PRP into the renal parenchyma during vascular clamping. The product would have been ineffective as a scaffold or a coat applied over the renal capsule. Systemic application would not have been effective since platelet concentrate is diluted in the bloodstream. In order to identify the possible effect of increased intrarenal hydrostatic pressure due to volume injection, we decided to compare PRP-treated subjects with a sham group in which the same volume of normal saline was injected.

Some hypotheses could explain why PRP worsened the kidney instead of regenerating it. The first one would be thrombi formation in intrarenal blood vessels. However, we did not find thrombi on histological examination one week after ischemia. Nevertheless, we cannot rule out early thrombus producing renal damage in the first days after ischemia. The second hypothesis is a greater renal damage due to the higher osmolality of PRP compared to saline solution. We cannot exclude this possibility; therefore a new study analyzing the osmolality of PRP and injecting a saline solution with the same osmolality in the sham group should be carried out to test this hypothesis. A third hypothesis is that the injected platelets also released cytokines and other proinflammatory agents, along with growth factors. It is well known that PRP releases inflammatory cytokines, especially when containing leukocytes. In fact, several studies have demonstrated a positive correlation between increasing leukocyte concentration in platelet concentrates and elevated levels of the inflammatory cytokines interleukin-1β (IL1β), tumor necrosis factor-α (TNF-α), interleukin-6 (IL-6), and interleukin-8 (IL-8) [46, 47]. In our study, PRP contained a mean of 5300 ± 3600/μL leukocytes, which could contribute to increased inflammatory response, worsening organ quality and producing kidney damage instead of tissue regeneration. There are several studies demonstrating that leukocyte depletion improves renal function after ischemia-reperfusion injury [48–51]. According to these authors, the principle behind the use of leukocyte depletion lies in the ability of activated leukocytes to promote or enhance the injury sustained by ischemic damage. Activated leukocytes increase oxidative stress by releasing oxidant compounds and inflammatory cytokines, decreasing anti-inflammatory counterparts, and activating several adhesion molecules that contribute greatly to cell and organ ischemic injury. However, there are many studies demonstrating the benefits of PRP containing white blood cells (WBC) [26, 27, 52–56]. Some authors state that PRP products containing WBC have been shown to inhibit the growth of some bacteria and improve healing in soft tissue injuries complicated by infection [27, 57]. However, the efficacy of WBC in PRP treatment remains unclear and may depend on indication. De Long et al. [28] suggest that PRP used to treat open wounds and prevent infection may require supranormal WBC levels, whereas PRP used to minimize scar formation should not contain WBC. Our study could be repeated using PRP without WBC to rule out an inflammatory reaction caused by the product as the cause of renal damage. A fourth hypothesis is that PRP could produce an immune reaction caused by the injection of a heterologous substance obtained from another rat. However, many other studies have used outbred rats, such as Sprague-Dawley or Wistar, in PRP studies without, apparently, altering the results [23, 58, 59]. We cannot therefore be sure that at least part of the damage was caused by an immune reaction. It would be better for future studies to use inbred rats, applying PRP to an immunologically identical animal, and thereby avoiding an immune response. A fifth hypothesis is that the method of administration, injecting the product while kidneys were still ischemic, may cause a compartment syndrome, and this must have induced increased intracellular pressure, enhanced interstitial edema and cytokine release in ischemic tissues. However, the same amount of product was injected in both groups, so the compartment syndrome and damage would have occurred equally in both groups. The last hypothesis that could explain the treatment failure in our study is that the application of PRP may be inefficient in a solid organ, and unlike nonsolid organs it might not operate correctly at the local level. However, the fact that PRP kidneys showed greater deterioration than sham kidneys demonstrates that there is a local effect of PRP, although that effect is not beneficial.

Our results may also have been influenced by the timing of PRP application. It would be interesting to study whether the application of PRP after relief from ischemia decreases kidney damage.

In any case, our results show that PRP type P4-x-Bβ applied subcapsularly in rat kidney subjected to ischemia-reperfusion, not only fails to improve the quality of the organ, but actually deteriorates it. This unexpected result should be tested using other PRP types. It would be desirable to test other forms of application as well; we ruled-out intra-arterial application during ischemia because intrarenal thrombi could be expected, adding damage factors to the supposedly beneficial effect of PRP.

One of the limiting factors of our study was the size of the animals, which did not allow us to perform serial creatinine and urea determinations without leading to anemia. It would have been interesting to take several determinations of creatinine and urea and analyze the exact effect of PRP on kidney function over time, presenting its effect without bias. Another limiting factor was the time of euthanasia, which was established at day seven after renal ischemia. While this allowed us to carry out more complete biochemical, histological and vascular flow studies, since a greater number of rats were alive at the end of the study, this limit did not allow us to conduct a long-term survival study or analyze the long-term effects of PRP on kidneys.

## Conclusions

This study shows a deterioration of the quality of rat kidneys treated with P4-x-Bβ platelet-rich plasma during ischemia-reperfusion. This damage was objectivized directly by histology and indirectly by ultrasound renal flow parameters. Also, we found correlations between these variables and kidney weight, kidney volume, and serum levels of creatinine and urea that support this conclusion.
